# Heterogeneity in Flood Hazard Exposure in 363 Latin American Cities

**DOI:** 10.34133/ehs.0567

**Published:** 2026-07-10

**Authors:** Jinwen Xu, Iryna Dronova, Vicente Tinoco, Josiah L. Kephart, Usama Bilal, Brisa N. Sanchez, Ana V. Diez Roux, Nelson Gouveia, Olga L. Sarmiento, Alejandra Vives, Marcio Alazraqui, Daniel A. Rodriguez

**Affiliations:** 1 Institute of Transportation Studies, University of California, Berkeley, Berkeley, CA 94720, USA; 2 Department of Landscape Architecture and Environmental Planning, University of California, Berkeley, Berkeley, CA 94720, USA; 3 Department of Environmental Science, Policy and Management, University of California, Berkeley, Berkeley, CA 94720, USA; 4 Urban Health Collaborative, Drexel Dornsife School of Public Health, Philadelphia, PA 19104, USA; 5 Department of Environmental and Occupational Health, Drexel Dornsife School of Public Health, Philadelphia, PA 19104, USA; 6 Department of Epidemiology and Biostatistics, Drexel Dornsife School of Public Health, Philadelphia, PA 19104, USA; 7 University of São Paulo Medical School, São Paulo 01246-903, Brazil; 8 School of Medicine, Universidad de los Andes, Bogotá 111711, Colombia; 9 School of Public Health, Pontificia Universidad Católica de Chile, Santiago 8331150, Chile; 10 Instituto de Salud Colectiva, Universidad Nacional de Lanus, Buenos Aires B1826GLC, Argentina; 11 Department of City and Regional Planning, University of California, Berkeley, Berkeley, CA 94720, USA

## Abstract

Flooding is among the costliest natural disasters worldwide, yet how riverine flood hazard is distributed within cities remains poorly quantified, particularly across socially vulnerable population groups. Climate change is intensifying flood magnitude, duration, and frequency, raising concerns that existing inequalities in flood exposure will widen. Using data from the multi-country *Salud Urbana en América Latina* project, we assess disparities in riverine flood hazard among residents of 363 cities across 9 Latin American countries. We develop 2 city-level indicators, the exposure deviation index (*EDI*) and the *Gini_flood_* index, to quantify disproportionality and spatial unevenness in populations residing in riverine flood-prone areas. Across 297.9 million urban residents, 6.9% are exposed to floods with an annual exceedance probability of 0.2% (500-y events), and children (<10 y) are more exposed than others. *EDI* values are generally small and slightly negative at the country level, indicating that populations are not systematically overrepresented or substantially underrepresented in hazard areas relative to land share of flood-prone area extent, although substantial variation exists across cities and countries. In contrast, *Gini_flood_* values are consistently high, indicating that exposed populations are strongly concentrated within a limited number of spatial units. Inland cities at higher elevations tend to exhibit greater disproportionality and unevenness, while at the regional scale, differences across age and education subgroups are limited. These findings provide transferable indicators for a relative assessment of flood hazard heterogeneity and underscore the need for equitable land use, housing policy, and infrastructure planning to advance climate resilience and environmental justice.

## Introduction

Flooding causes tens of billions of dollars in direct economic losses annually and has become one of the costliest natural disasters worldwide [1]. Currently, more than 1.81 billion people, over 1 in 5 globally, are directly exposed to a 1-percent-annual-chance flood [2]. Extreme weather events and rising sea levels driven by climate change are projected to increase the severity, duration, and frequency of floods [3–5]. By 2100, under a “middle-of-the-road” (Socioeconomic Pathway 2-4.5) climate scenario, the number of people living in areas exposed to 1-percent-annual-chance floods is projected to be 15% to 20% higher than at present [6]. Flood risks, however, are not distributed equally among the population and have many impacts on human health and wellbeing beyond financial loss. Disadvantaged populations are found to be more likely to reside in flood-prone areas, due in large part to historical settlement patterns, low income, limited land and housing options, and other socio-environmental reasons [7–10]. The degree of differential exposure among disadvantaged populations makes them more vulnerable to the immediate impact of floods and creates additional challenges for long-term recovery [11,12].

Latin American cities, marked by high urbanization [13], socioeconomic segregation [14], income inequality [15], and high exposure to climate-related hazards [16–18], are critical contexts where inequalities in flood exposure are likely to exacerbate environmental injustices. Though various aspects of the urban environment have been associated with flood hazards, it remains unclear which specific features account for differences among cities in the region [19], and limited understanding of the interactions between flood hazard, at-risk populations, social disparities, and the built environment remains a barrier to effective climate adaptation planning [16,18]. Moreover, there is no consensus on how to define and operationalize flood hazards for equity analyses, leading to inconsistent risk metrics and a lack of standardized ways to measure disparities across population subgroups. Although some studies have examined the occurrence of floods and flood hazards and associated social disparities in Latin American cities [18,19], the heterogeneity in population and subgroups prone to flood hazards has not been systematically addressed.

Heterogeneity in population exposure to riverine flood hazards can be characterized in at least 2 ways: (a) inequalities in exposure can be assessed by comparing the share of the population living in flood-prone areas to the share of the city area that is flood-prone across various social groups, and (b) spatial heterogeneity or unevenness in exposure can be assessed by comparing the cumulative shares of population and cumulative shares of flood-prone areas across spatial units (using a modified version of the *Gini* coefficient). The first assesses whether particular groups are disproportionately represented in flood-prone areas relative to the land area at risk, whereas the second captures both the degree of population exposed to flood-prone areas and how evenly or unevenly the population is distributed across flood-prone areas. Empirical evidence supports the presence of both types of heterogeneity in exposure to flood hazards [18,20]. Prior work shows that certain population subgroups, such as disadvantaged residents, are often more disproportionately exposed to flood risks [7,21], but limited work has focused on Latin American cities.

By overlaying flood hazard maps, building-footprint data, and harmonized population data across 363 Latin American cities, we estimate total and subgroup populations exposed to flood hazards using the global riverine flood hazard maps from the European Commission Joint Research Centre (JRC). We then calculate 2 indices that measure heterogeneity in exposure, as described above, one that quantifies disproportionality and another that measures unevenness in flood hazards for the overall population and for population subgroups. Together, these indices provide actionable metrics for urban risk management by identifying priority areas for targeted intervention and highlighting patterns relevant to environmental justice. Next, we examine associations between these indices and local social and environmental conditions for each city. This framework standardizes measurement and improves comparability across cities. Our research questions are as follows:

- How disproportionate is the exposure to riverine flood hazard relative to the share of city land within flood-prone areas across the overall population and population subgroups?
- How even is the spatial distribution of the population (as a whole and population subgroups) across riverine flood hazard areas?
- Which social and environmental features of cities are associated with higher city-level disproportionality and unevenness of riverine flood hazard exposure?

We hypothesize that older adults, children, and people with lower educational attainment are more disproportionately exposed to riverine flood hazards (henceforth referred to as flood hazards) relative to the share of city land within flood-prone areas than their counterparts. Similarly, we hypothesize that populations are unevenly distributed across spatial flood hazard areas. Finally, we expect cities characterized by rapid population growth, high population density, dense transportation networks, higher economic activity, and challenging physical environments (e.g., high altitude, steep slopes, rough terrain, limited green space, or coastal location) to exhibit greater disproportionality and unevenness in population exposure to flood risk.

## Materials and Methods

### Study area and city selection

This study is conducted as part of the *Salud Urbana en América Latina* (SALURBAL) project, an international collaboration that has compiled and harmonized data on social, environmental, and health characteristics for all cities with at least 100,000 residents in 2010 across 11 Latin American countries (*n* = 371). SALURBAL defines cities as clusters of municipalities that cover the built-up extent of urban areas, as identified from satellite imagery and further local consultation [22]. This definition captures a wide range of urban areas, from small cities to megacities. Population counts within these cities are obtained from national censuses and organized into small-area geographic units at the neighborhood level, broadly comparable to US census tracts (Table S1). The naming and definitions of these units vary by country, with details provided in Table S1. We excluded 8 cities in El Salvador and Nicaragua due to missing census population data at the neighborhood level. The final sample includes 363 cities located in Argentina, Brazil, Chile, Colombia, Costa Rica, Guatemala, Mexico, Panama, and Peru.

### Flood hazard maps

We use the European Commission JRC global flood hazard maps to represent flood-prone areas across the 363 Latin American cities [23]. These maps provide global gridded inundation estimates for multiple return periods (e.g., 10, 20, 50, 100, 200, and 500 y) at 90-m resolution, which can be directly linked to annual flood probabilities and allow sensitivity analysis across different return periods. The dataset is generated using a coupled hydrologic and hydrodynamic modeling framework that integrates the LISFLOOD rainfall-runoff model with the LISFLOOD-FP inundation model to simulate river discharge and flood-prone area extent at the global scale [23–25]. It has been validated with official flood hazard maps and satellite-derived flood maps and shows adequate performance [23]. These flood hazard maps have been widely used in global and regional assessments of riverine flood exposure and risk due to their consistent methodology and explicit representation of flood frequency [5,26,27]. In this study, we use the 500-year return period to represent rare and large riverine floods as the primary hazard scenario, while we use other return periods to evaluate the sensitivity of exposure patterns to flood frequency. Following prior work [28,29], we interpret inundated areas under each return period as the spatial extent of the flood hazard at that frequency.

### Population exposed to flood hazards

We apportion population data to a uniform grid using building footprints and overlay the hazard maps to calculate the proportion of inundated areas within each cell. Specifically, we use neighborhood-level population counts from each country’s population census in the latest available year (Table S1) for total population (Total), children aged less than 10 years (<10 y), young and middle age residents (10 to 64 y), older adults aged 64+ years (>64 y), adults aged 25+ who completed primary education or more (>25 y w/ primary education), and adults aged 25+ who did not complete primary education (>25 y w/o primary education). To apportion population to grid cells, we first construct a regular grid of 0.01° cells, approximately 1-km resolution (ranging from 0.725 km^2^ near the pole to 1.231 km^2^ near the equator due to geographic projection distortion) for the 363 cities and retained all cells intersecting city administrative boundaries. Then, we use building footprints, obtained from the Google Open Buildings dataset [30], to calculate the proportion of building-footprint area of each census unit that falls within each cell and allocate the population based on that proportion. Exposed populations in each cell are aggregated to the city and country levels for further description. We also group grid cells into quintiles based on grid-cell population percentages of children aged less than 10 years, older adults aged 64+ years, and adults aged 25+ who completed primary education, using the full set of study grid cells to define the quintile cut points.

(1) $$P_{g}^{Flood-prone}=\underset{i}{\sum }\left(P_{i}\times \frac{\sum_{b\in i\cap g}Area_{b}^{Flood-prone}}{\sum_{b\in i}Area_{b}}\right)$$

where $P_{g}^{Flood-prone}$ is the population in flood-prone areas of grid cell *g*, *P_i_* is the total population of neighborhood *i*, $Area_{b}^{Flood-prone}$ is the area of building footprint *b* that lies in flood-prone areas within grid cell *g*, $\sum_{b\in i\cap g}Area_{b}^{Flood-prone}$ represents the total area of flood-prone building footprints from neighborhood *i* inside grid cell *g*, and ∑_*b*∈*i*_ *Area*_*b*_ is the total building footprint area in neighborhood *i*.

### Indices of flood hazards

To assess disproportionate and uneven distribution of population exposed to flood hazard, we use 2 indices that reflect (a) the proportion of the city population that lives in flood-prone areas relative to the share of city land that is flood-prone, and (b) how evenly the population is distributed across cells with varying levels of flood-prone area coverage. The first index, the exposure deviation index (*EDI*), measures whether population (or subgroups) are disproportionately located in flood-prone areas, that is, whether the observed population share in flood-prone areas exceeds what would be expected based on the city’s share of land in flood hazard areas. The second (the *Gini_flood_* index) summarizes the unevenness of the distribution of population (or subgroups) exposed to flood-prone areas across grid cells, indicating the extent to which exposed populations are concentrated in specific locations within a city.

### Exposure deviation index

The *EDI* is calculated for each city as the difference between the proportion of population living in flood-prone areas (*U*^*City*^, see Eq. 2) and the proportion of land area that is flood-prone (*L*^*City*^, see Eq. (3)), as defined in Eq. (4). This index is helpful because cities differ widely in the share of their flood-prone areas, and therefore direct comparisons of raw flood risk across cities may be misleading. For example, coastal or riverine cities may have up to 90% of their land classified as flood-prone and, consequently, a larger share of their population exposed, whereas inland or arid cities may have only a small fraction of flood-prone land and a lower baseline level of exposure (see Fig. S1). *EDI* quantifies how much of a city’s flood-prone population share deviates from what would be expected given its flood-prone extent. The expected value of *EDI* ranges from –1 to +1, where negative values indicate underrepresentation of population in flood-prone areas and positive values indicate overrepresentation of population in flood-prone areas. Both extremes reflect disproportionality, but a negative *EDI* is preferred since it implies a lower share of people residing in flood-prone areas. An *EDI* of 0 denotes proportionality, meaning the share of population in flood-prone areas is equal to the share of land in flood-prone areas. Statistical significance of *EDI* is evaluated using a Monte Carlo permutation test, as described in the “Statistical analysis” section.

(2) $$U^{City}=\frac{\sum_{g\in City}P_{g}^{Flood-prone}}{\sum_{g\in City}P_{g}}$$

(3) $$L^{City}=\frac{Area_{City}^{Flood-prone}}{Area_{City}^{Administrative-boundary}-Area_{City}^{Existing-water-bodies}}$$

(4) $$\begin{array}{l} EDI^{City}=U^{City}-L^{City}=\frac{\sum_{g\in City}P_{g}^{Flood-prone}}{\sum_{g\in City}P_{g}}\\ -\frac{Area_{City}^{Flood-prone}}{Area_{City}^{Administrative-boundary}-Area_{City}^{Existing-water-bodies}} \end{array}$$

where *P_g_* is population in grid cell *g*, ∑_*g* ∈ *City*_*P*_*g*_ is the total population of grid cells in the given city, and $\sum_{g\in City}P_{g}^{Flood-prone}$ is the flood-prone population in the city.

We also calculate the *EDI* for specific population subgroups based on age and educational levels described in the previous section. We examine the distribution of subgroup *EDI* values at the city level and compare these patterns across countries.

### Gini_flood_ coefficient

**Fig. 1:**
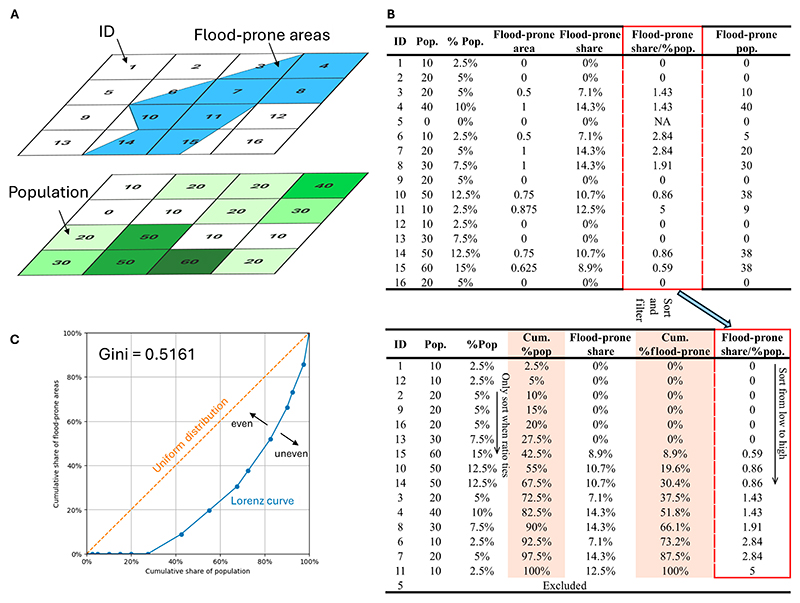
Lorenz curve and *Gini_flood_* coefficient using fictitious population and flood-prone area: (A) spatial distribution of population and flood-prone areas at grid-cell level; (B) raw records (top) and the same records sorted by share (highlighted in red dashed boundary); (C) Lorenz curve (built from highlighted records in panel B with cumulative population share in *x*-axis and cumulative flood-prone area share in *y*-axis) with the line *y* = *x* indicating a uniform distribution and the *Gini_flood_* computed from data in panel B. More even flood-prone exposure is reflected when the Lorenz curve approaches the equality line, whereas greater unevenness occurs as the curve deviates further away from it; NA stands for not available.

The second index is a summary measure of the proportion of population living in flood-prone areas and the spatial unevenness in population exposure to flood hazards (Fig. 1). We develop this indicator by modifying the *Gini* index [31] and referring to our indicator as the *Gini_flood_* coefficient. For each city, we rank each grid cell that has flood-prone areas inside from low to high based on the ratio of its flood-prone area share to its population share (relative to the total city flood plain area and city population, respectively). This ranking is used to derive the cumulative distribution of flood-prone areas across the flood-prone population (Fig. 1B). Ties in the flood-prone area-to-population ratio are resolved by ranking spatial units according to their population share, also in ascending order. The Lorenz curve is then constructed from the ordered spatial units (Fig. 1C). Equation (5) is used to calculate *Gini_flood_* (in the middle part of Eq. (5)), and the formula can be further simplified to the final format on the right:

(5) $$GINI_{flood}=\frac{Area_{Between-Lorenz-and-equality-line}}{Area_{Under-equality-line}}=\frac{\sum_{i=1}^{n}(2i-n-1)x_{i}}{n\sum_{i=1}^{n}x_{i}}$$

where *Area*_*Between*−*Lorenz*−*and*−*equality*−*line*_ represents the area between the Lorenz curve and the equality line, *Area*_*Under*−*equality*−*line*_ represents the area under the equality line (normal distribution of population in flood-prone areas), *x* represents the share of population (or subgroups) living in flood-prone areas in spatial unit (sorted in ascending order), *n* is the total number of spatial units nested within each city, and *i* is the rank of each *x* value (population share) sorted in ascending order within each city.

We calculate *Gini_flood_* for both the total population and population subgroups. Specifically, *Gini_flood_* is a summary measure constructed by calculating 2 variables for each grid cell: (a) the proportion of the city population that lives in the grid cell and (b) the proportion of the city’s total flood-prone area contained in that cell. The *Gini* is calculated by plotting the cumulative proportion of flood-prone areas in the grid cells (*y*-axis) versus the cumulative population proportion in the grid cells (*x*-axis). If most of the population resides outside flood-prone areas (i.e., many grid cells with zero flood-prone area), the initial segment of the Lorenz curve will be flat and lie along the *x*-axis (i.e., increasing amounts of cumulative population will live in grid cells with 0% cumulative flood-prone areas), producing a relatively high *Gini_flood_* value. Beginning with the first grid cell containing any flood-prone areas, the remaining curve is constructed by ranking the ratio of population share to flood-prone area share from low to high. A greater divergence between their cumulative distributions and the line of uniform distribution indicates stronger spatial unevenness in flood-prone area exposure. *Gini_flood_* ranges from 0 to 1, where higher values denote both a smaller population living in flood-prone areas and greater spatial unevenness of exposure (i.e., exposed residents are concentrated in a few grid cells), while lower values indicate a more even distribution of flood risk across the city. If cumulative population and cumulative flood-prone area shares are perfectly aligned, the Lorenz curve will coincide with the line of equality (*y* = *x*), yielding a *Gini_flood_* coefficient of 0. An example of detailed population and percentage of flood-prone areas at grid-cell levels is prepared for the calculation of *Gini_flood_* in Fig. 1.

### City characteristics associated with heterogeneity

We use data on 9 urban environmental variables from the SALURBAL study, including population growth from 2015 to 2020, population density, road intersection density, GDP per capita, average elevation, slope, standard deviation of slope, green space, and coastal location. Appendix SI provides the definitions, data sources, and reference years for each urban environmental variable.

### Statistical analysis

To evaluate whether the observed *EDI* differs significantly from what would be expected under a random spatial distribution of population given the same flood risk level, we perform a Monte Carlo permutation test. For each city, we randomly permute the total population among all grid cells within the city 999 times while keeping the flood-prone areas for each cell fixed. For each permutation, we re-calculate the *EDI*, generating a null distribution of *EDI* values that reflects the scenario where there is no spatial association between population and flood risks. The observed *EDI* is then compared to this simulated null distribution. We compute a *P* value as the proportion of permutations in which the absolute value of the simulated *EDI* is greater than or equal to the absolute value of the observed *EDI*. We also calculate a *z*-score by comparing the observed *EDI* to the mean and standard deviation of the simulated values. All simulations and calculations are repeated for all 363 cities. Furthermore, we plot histograms comparing cities with significant and nonsig-nificant *EDI* by the proportion of flood-prone areas within their administrative boundaries. This step controls the underlying extent of flood-prone areas, ensuring that observed differences in *EDI* significance are not simply an artifact of cities with larger flood-prone areas appearing more likely to yield significant results. Since *Gini_flood_* lacks clear benchmark values, developing a statistical significance test is not meaningful; therefore, we apply the Monte Carlo permutation test only to *EDI*.

To assess whether *EDI* and *Gini_flood_* values differ across countries, we apply Kruskal–Wallis tests to the distributions of each index by country/region. We further conduct pairwise boot-strap analyses (5,000 resamples) to estimate 95% confidence intervals for the mean differences between region pairs and identify specific country contrasts that contribute to between-country variation.

We conduct a univariate city-level correlation analysis between the 2 indices across age and education groups and the 9 urban environmental variables using the Spearman correlation coefficient. For all cities, we also plot their *Gini_flood_* against *EDI* values to discern common patterns and classify them into 4 quadrants representing distinct combinations of flood risk disproportionality and unevenness.

## Results

### Populations living in flood-prone areas in 363 cities

Among the 297.9 million residents, 20.6 million (6.9%) live in flood-prone areas (Table 1). There is substantial variation across countries in the proportion of urban residents living in flood-prone areas. In Argentina, almost 20% of residents live in flood-prone areas, while cities in Central America, Chile, Colombia, and Peru have less than 5% live in flood-prone areas. Mexico (6.9%) and Brazil (5.2%) have an intermediate share of its population living in flood-prone areas, but the number of flood-prone population (6.7 million and 5.7 million, respectively) is the highest among all countries. At the city level, Resistencia (93%) and Santa Fe (65%) in Argentina and Macapa (91%), Itajai (75%), and Rio Grande (72%) in Brazil have the highest proportion of residents living in flood-prone areas. Owing to their large population size, Buenos Aires in Argentina (3.9 million), Mexico City (2.7 million) in Mexico, Bogota in Colombia (0.8 million), and Sao Paulo (0.6 million) and Porto Alegre in Brazil (0.5 million) are the cities with the highest number of residents living in flood-prone areas.

For population subgroups, children aged <10 years (7.3%) show a slightly higher share of residing in flood-prone areas than other groups (Table 1). This pattern is evident in cities in Argentina, Brazil, Colombia, and Peru. In contrast, cities in Argentina, Colombia, and Peru show markedly lower proportions of older adults living in flood-prone areas relative to other age groups. Additionally, in most countries except Mexico and Central America, a larger proportion of adults who did not complete primary education reside in flood-prone areas compared to those with primary education, with Argentina and Peru showing the most pronounced differences.

### Exposure deviation index

**Fig. 2:**
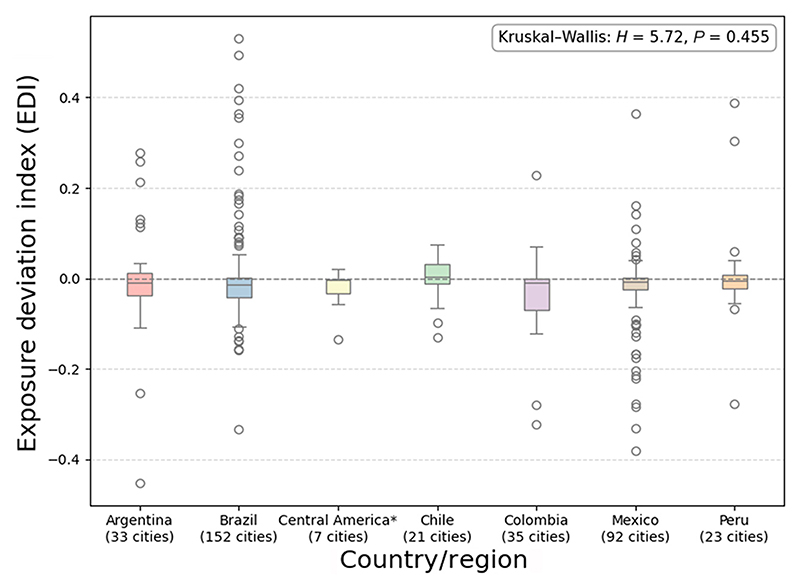
Distribution of the exposure deviation index (*EDI*) using total population by country/region with the gray lines in each box representing median *EDI* values, and boxes spanning the 25th to 75th percentiles (interquartile range); Kruskal–Wallis test results are annotated in the upper-right corner of the panel.

**Fig. 3:**
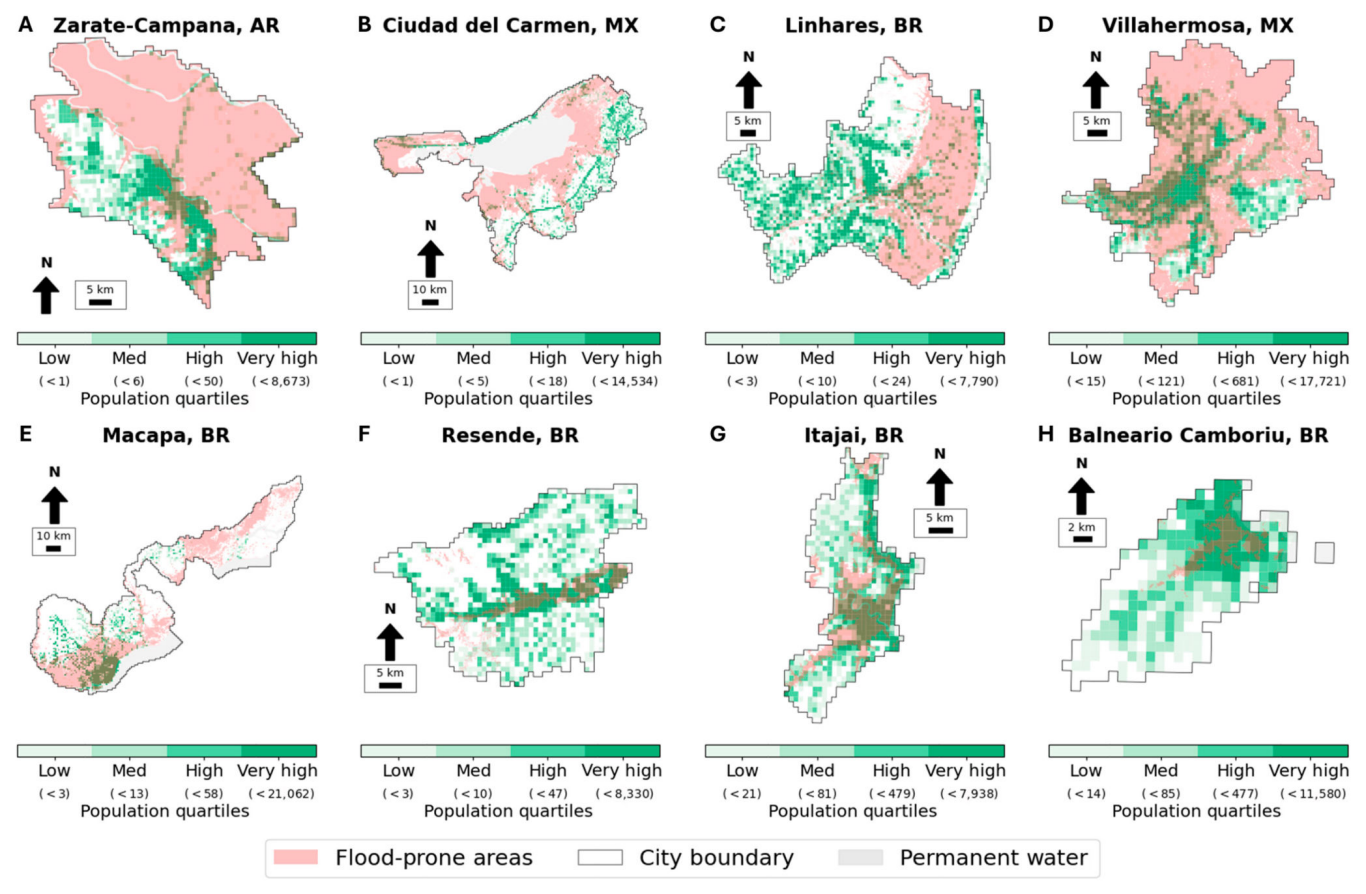
0.01° grid-cell level (~1 km) population count (approximately equivalent to population density) and flood-prone area distributions in the metropolitan area with the 4 highest and 4 lowest *EDI* values: (A) Zarate-Campana in Argentina (*EDI* = –0.45), (B) Ciudad del Carmen in Mexico (*EDI* = –0.38), (C) Linhares in Brazil (–0.33), (D) Villahermosa (–0.33) in Mexico, (E) Macapa in Brazil (+0.53), (F) Resende in Brazil (+0.49), (G) Itajai in Brazil (+0.42), and (H) Balneario Camboriu in Brazil (+0.40), with population grouped by 4 classes (quantiles).

Of the 363 Latin American cities, most have an *EDI* ranging from approximately –0.2 to +0.2 (Fig. 2). Country-level differences are not significant overall (Kruskal–Wallis *P* = 0.455), though Brazilian cities show slightly higher *EDI* than those in Colombia and Mexico (Fig. S2). A majority of cities (72.5%, 263 out of 363) have negative *EDI*s, suggesting that more cities have their residents likely to reside outside flood-prone areas than expected if the population is randomly distributed across the city. Cities with the most negative *EDI*, such as Zarate-Campana (–0.45) in Argentina, Ciudad del Carmen (–0.38) and Villahermosa (–0.33) in Mexico, and Linhares (–0.33) in Brazil, demonstrate that residents of these cities are less likely to live in flood-prone areas than expected if the population are randomly distributed across the city (see the top panels of Fig. 3). In contrast, 4 cities in Brazil, Macapa (+0.53), Resende (+0.49), Itajai (+0.42), and Balneario Camboriu (+0.40), have the highest positive *EDI*, indicating a greater likelihood of residents living in flood-prone areas (see the bottom panels of Fig. 3).

The distributions of *EDI* for the age and education subgroups examined are approximately symmetric and centered slightly below zero (median = –0.008), with most cities exhibiting values close to zero (Fig. S3). The *EDI* for subgroups is consistent with those of the total population, indicating similar patterns of disproportionality across all age and education groups.

Aggregated at the country level, the distribution of the *EDI* reveals substantial variation across Latin American countries (Fig. 2). Most countries exhibit negative median *EDI* for the total population, with Brazil (median *EDI* = –0.014), Argentina (median *EDI* = –0.009), and Colombia (median *EDI* = –0.009) showing the lowest values, while Chile is the only country with a positive median *EDI* (+0.003). In terms of variation across cities, Colombia (interquartile range [IQR] = 0.067) exhibits the greatest dispersion in *EDI* among all countries.

Based on Monte Carlo permutation tests, of the 363 cities analyzed, 155 have significant *EDI* at *P* <0.05, including 64 positive and 91 negative cases (Table S2). Across population subgroups, the number of cities with positive and significant *EDI* is slightly higher for children compared to the total population (70 vs. 64), and cities with negative and significant *EDI* among older adults are also fewer than for the total population (79 vs. 91). Country-level tests reveal broadly consistent patterns, with the number of cities in each significant category differing from the total population by only a few cases.

To examine whether results are influenced by the proportion of flood-prone area extent, we compare cities with significant and nonsignificant *EDI* using histograms of the proportion of riverine flood-prone areas within each city’s boundary (Fig. S4). The distributions show no clear pattern, indicating that significance is not confined to cities with any particular proportion of flood-prone areas.

### Unevenness in the distribution of flood risk (*Gini_flood_*)

**Fig. 4:**
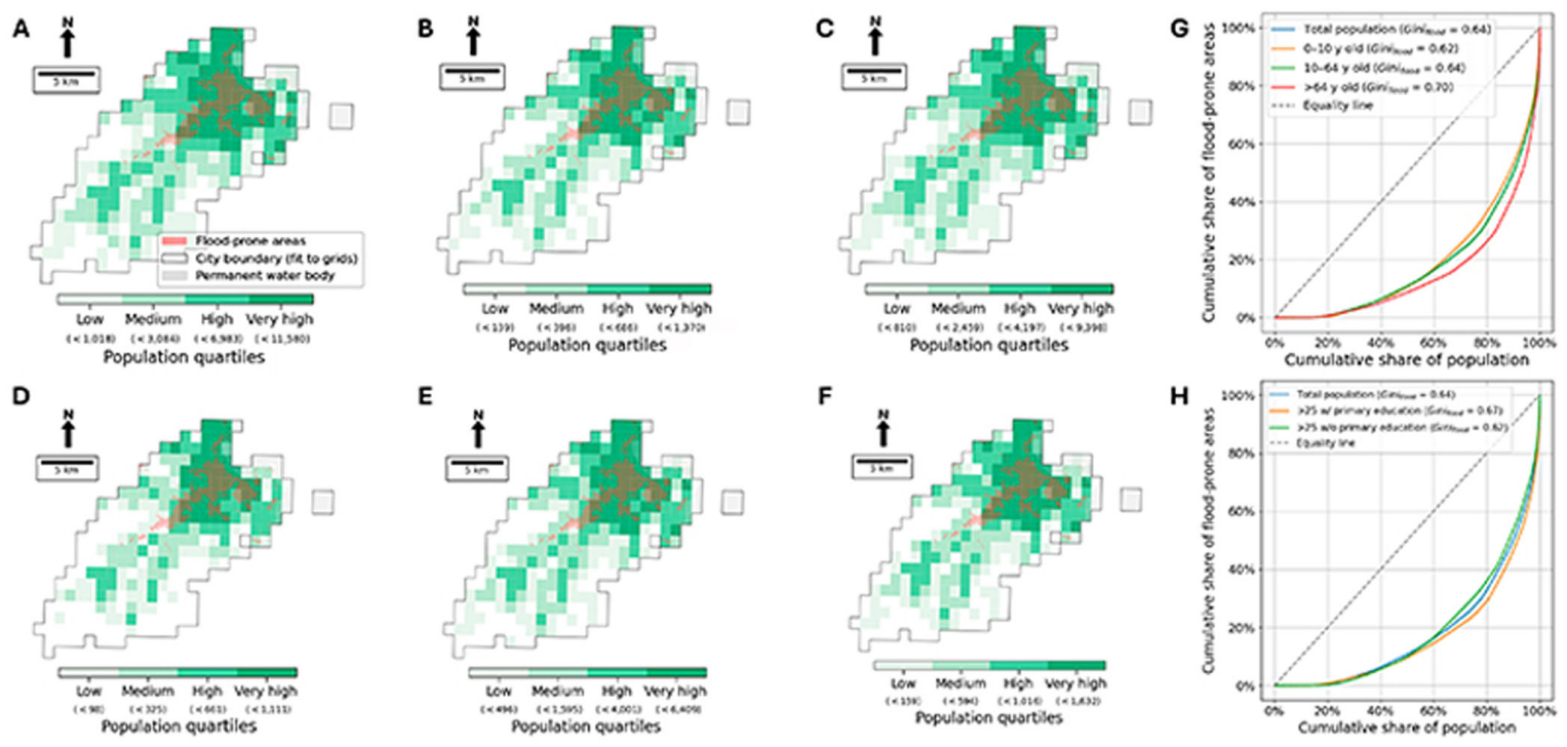
Spatial distribution of total population and subgroups at the grid-cell level in Balneario Camboriu, Brazil, which is selected since the city has the largest variation in *Gini_flood_* across population subgroups, and corresponding Lorenz curves by quartile for (A) total population, (B) children (<10 y old), (C) young and mid-age people (10 to 64 y old), (D) elderly (>64 y old), (E) adults aged 25+ who completed primary education or more, (F) adults aged 25+ who did not complete primary education, and Lorenz curve of total population with (G) age subgroups and (H) education subgroups showing the cumulative share of flood-prone areas shared by population living in each unit, compared with *y* = *x* equality line (dashed gray line) which indicates uniform distribution.

*Gini_flood_* values range from 0.64 to 1, with a majority cities (78.9%) exhibiting values above 0.95 which indicate that most of city population live outside flood-prone areas and a small share of the population is concentrated in cells with a high share of the flood-prone areas (see distribution of *Gini_flood_* in Fig. S5). Two cities are excluded from this calculation as no flood-prone areas are identified within their boundaries, which are Cusco in Peru and Teziutlan in Mexico. The *Gini_flood_* for adults who did not complete primary education show the greatest variation across cities (IQR of *Gini_flood_* = 0.045) while adults who completed primary education (median *Gini_flood_* = 0.983) and the older adults (median *Gini_flood_* = 0.982) subgroup have a higher median value of *Gini_flood_* than other subgroups. Figure 4 illustrates the grid-cell level spatial distribution of population in flood-prone areas and the Lorenz curve used to calculate *Gini_flood_* in Balneario Camboriu, Brazil, which displays the greatest variation in *Gini_flood_* across all subgroups and is also the city with minimum *Gini_flood_*.

**Fig. 5:**
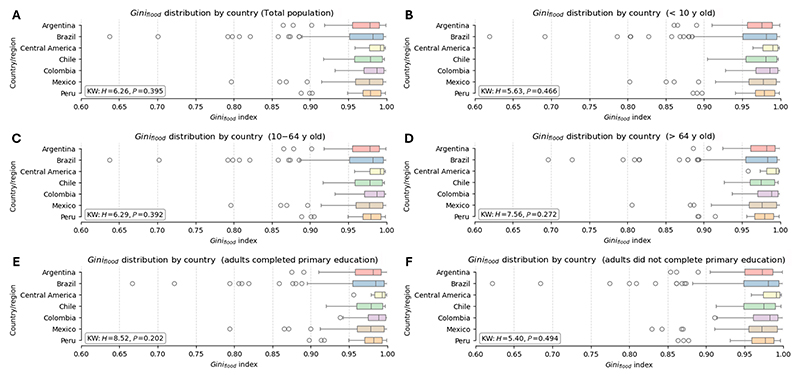
Distribution of *Gini_flood_* for (A) total population and subgroups: (B) <10 years old, (C) 10 to 64 years old, (D) >64 years old, (E) adults who completed primary education, and (F) adults who did not complete primary education. Boxes span the interquartile range (25th to 75th percentiles) with gray lines indicating medians; Kruskal–Wallis (KW) test results are annotated in the lower-left corner of each panel.

At the country level, the unevenness of the population distribution in flood-prone areas varies, with a majority of *Gini_flood_* values for the total population above 0.95 (79%, 285 out of 361; see Fig. 5). Differences across countries are not statistically significant (Kruskal–Wallis *P* > 0.2 for all population subgroups), though pairwise bootstrap analyses reveal modest differences between several specific country pairs (Fig. S6). Central America (median *Gini_flood_* = 0.992), Colombia (0.987), and Brazil (0.982) all show pronounced spatial heterogeneity, exhibiting either low percent populations in flood-prone areas or uneven population distributions within flood-prone areas. Brazil displays the greatest variation in *Gini_flood_* (IQR of *Gini_flood_* = 0.044), indicating larger differences among cities compared with other countries. Age and education subgroups largely mirror the overall *Gini_flood_* pattern across countries, with modest deviations: In Central America, elderly populations and adults with primary education are more unevenly distributed in flood-prone areas than the total population.

### Association between heterogeneity indices and city characteristics

**Fig. 6:**
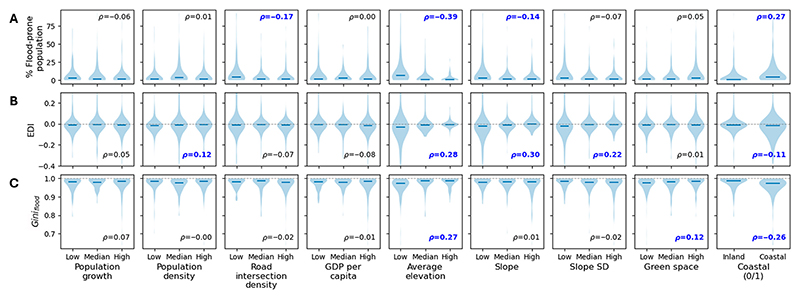
Grouped violin plots in tertiary quantiles (visualization purpose only) showing the relationships between the (A) share of population living in flood-prone areas, (B) *EDI*, (C) *Gini_flood_* index, and a range of socioeconomic and environmental variables across cities. Each cell displays the overall Spearman correlation coefficient (*ρ*), *ρ* with significant correlations (*P* < 0.05) highlighted in blue and bold font.

When examining associations between (a) the proportion of the flood-prone population, (b) *EDI*, and (c) the *Gini_flood_* coefficient, and a range of city-level socioeconomic and environmental factors (Fig. 6), we observe weak but statistically significant correlations across all 3 metrics. For the proportion of population living in flood-prone areas within the city, a significant positive association is detected for coastal locations (*ρ* = +0.27) and negative associations with road intersection density (*ρ* = −0.17), average elevation (*ρ* = −0.39), and slope (*ρ* = −0.14). Patterns of association estimated for age and education subgroups are similar with the total population (Fig. S7).

When considering relative exposure through the *EDI*, which accounts for the underlying share of flood-prone land within a city, we find it significantly and positively associated with population density (*ρ* = +0.12), average elevation (*ρ* = +0.28), mean slope (*ρ* = +0.3), slope standard deviation (*ρ* = +0.22), and coastal location (−0.11). This indicates that higher *EDI* values tend to occur in denser and inland cities at higher elevations with steeper and more heterogeneous terrain. Patterns of association for indices estimated for age and education sub-groups mirror the total population (Fig. S8). *EDI* is not associated with population growth, road intersection density, GDP per capita, green space, or coastal location.

*Gini_flood_* is negatively associated with coastal location (*ρ* = −0.26) and positively associated with elevation (*ρ* = +0.27) and green space (*ρ* = +0.12). A similar pattern of association holds when considering the heterogeneity indices for age and education subgroups (see Fig. S9). There is no association between *Gini_flood_* and population growth, density, road intersection density, GDP per capita, slope, or terrain roughness. Interpreting *Gini_flood_* as unevenness of at-risk population distribution, higher *Gini_flood_* tends to occur in inland cities with higher average elevation and more green space, whereas lower *Gini_flood_* is more common in lower, less green, and coastal cities.

In summary, although cities at lower elevations tend to exhibit higher overall flood exposure, disproportionate and uneven exposure is more pronounced in higher-elevation cities. A similar pattern is observed with respect to terrain: While a larger share of the population is exposed to flood hazards in low-gradient areas, individuals residing in steeper cities experience greater disproportionality in exposure. Coastal location shows a consistent trend. Despite higher aggregate flood exposure in coastal cities, inland cities display more pronounced spatial heterogeneity in exposure, with certain populations disproportionately and unevenly affected by flood hazards. Subgroup patterns mirror the total population with only minor variation, and none of the measures significantly relates to population growth or GDP.

### Joint assessment of indices

The values of the 2 indices for each of the 361 cities can be visualized when plotted together. Median values for each determine 4 quadrants, with cities falling into one of them. The distribution by quadrant is as follows: 6 cities (1.7%) in Quadrant I (+*EDI*, high *Gini_flood_*) with 3 cities having significant +*EDI*s, 174 cities (48.2%) in Quadrant II (−*EDI*, high *Gini_flood_*) with 59 cities having significant −*EDI*s, 88 cities (24.4%) in Quadrant III (−*EDI*, low *Gini_flood_*) with 32 cities having significant −*EDI*s, and 93 cities (25.7%) in Quadrant IV (+*EDI*, low *Gini_flood_*) with 61 cities having significant +*EDI*s.

**Fig. 7:**
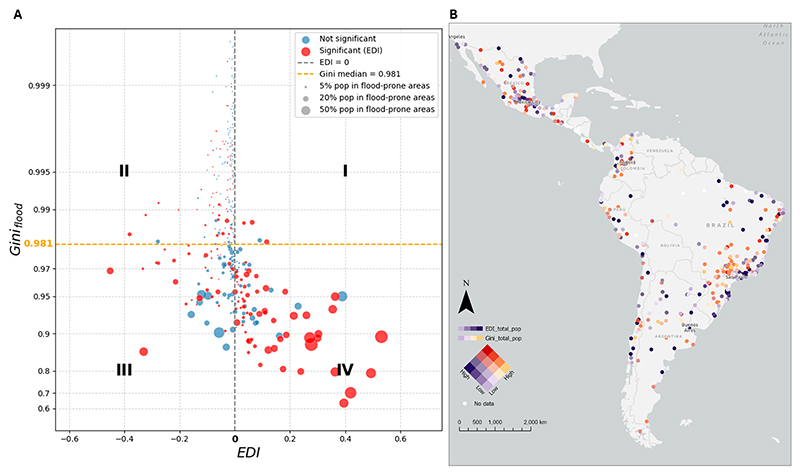
Classification of cities by *EDI* and *Gini_flood_*. (A) Scatterplot of *Gini_flood_* and *EDI*, with marker size proportional to the share of each city’s population residing in flood-prone areas. The *Gini_flood_* axis is transformed as −log(1 − *y*) for visualization purposes. Quadrant breakpoints selected at the median value for *Gini_flood_* and at 0 for *EDI*. (B) Bivariate choropleth map of 361 Latin American cities. All cities are classified into 4 quadrants according to the sign of *EDI* (>0 or <0; none equals 0) and whether *Gini_flood_* is above or below the median. Statistical significance of *EDI* is assessed using a Monte Carlo permutation test of random population distributions (*P* < 0.05). Cities with significant *EDI* are highlighted. The 4 quadrants represent (a) positive *EDI* + high *Gini_flood_*, (b) negative *EDI* + high *Gini_flood_*, (c) negative *EDI* + low *Gini_flood_*, and (d) positive *EDI* + low *Gini_flood_*.

The distribution across quadrants indicates a slightly negative linear association (Fig. 7A): As a higher proportion of people reside in flood-prone areas relative to the share of city land in flood-prone areas (positive *EDI*), the distribution of population flood risk becomes more even (lower *Gini_flood_*). The 3 Quadrant I cities with a statistically significant *EDI*, i.e., Parana (Argentina), Santa Marta (Colombia), and Rio Branco (Brazil), combine a higher share of the population residing in flood-prone areas relative to the city area in flood-prone areas with a highly uneven distribution of flood risks among residents. This warrants attention because the cluster of residents living in areas with a high share of the flood-prone areas may face elevated flood risks. The largest share of significant cases lies in Quadrant IV, indicating many cities where the share of flood-prone populations is higher than land share of flood-prone areas, yet population is distributed more evenly across cells with flood hazards. Quadrant II reflects a lower share of flood-prone residents relative to the share of city land in flood-prone areas but with concentrated and uneven pockets of flood-prone residents, whereas Quadrant III reflects the same comparatively lower share of residents in flood-prone areas and with relatively even hazard exposure. Spatially, many cities in Quadrant IV are concentrated along the coastlines of Brazil and Chile and in central Mexico, with additional cases in inland Argentina, whereas cities in Quadrant III are more commonly found in inland regions (Fig. 7B).

## Discussion

This study presents a city-level assessment of heterogeneity in flood hazards among 363 Latin American cities. To our knowledge, it is the first regional quantitative analysis of flood hazard disproportionality and unevenness as assessed by residence in flood-prone areas across a large set of cities. Following prior work [7,21,32], we quantify intra-urban variation in flood hazard exposure using 2 complementary indices, resulting in 6 key findings.

First, the findings suggest that urban flood hazards in Latin America are broadly comparable to global averages when consistent definitions are applied. At the country level, Argentina shows the highest proportion of urban residents living in flood-prone areas (around 20% in our definition), while Brazil and Mexico account for the largest populations in flood-prone areas. These patterns are consistent with prior studies identifying Brazil, Argentina, and Mexico as countries with elevated flood-related mortality and economic losses in the region [19]. We find that about 20.6 million people, 6.9% of the 297.9 million Latin American residents, live in areas under threat from flood hazards (defined as an annual exceedance probability of 0.2%, i.e., 500-y return period events). These estimates provide a baseline for preparedness and mitigation planning at national and regional scales. Previous studies have reported considerably higher exposure levels, ranging from 16.7% [18] to 23% [2]. These studies adopt different definitions of flood hazard and generally apply a binary definition of exposure, whereby an entire spatial unit (e.g., neighborhoods [18] or 90-m grid cells [2]) is considered exposed if any portion intersects with the flood extent. Although straightforward, this approach can overestimate exposure as it assumes uniform exposure within each unit. In contrast, our grid-cell-level estimates incorporate building-footprint-area weighting, allowing exposure to be allocated more precisely within each unit and yielding lower estimates. For comparison, we reconstructed exposure using the same binary approach for our grid cells. Under this definition, population exposed to flood hazards increases to 22.2% (Table S3), closely aligned with previous estimates. This confirms that differences are in part driven by how exposure is defined. Other methodological choices, such as characterizing exposure based on recorded presence of a flood within 2000–2018 as in Kephart et al. [18] versus estimated residential location within flood-prone area, may also contribute to these differences. Because our primary results are based on the 500-year flood extent, which represents rare but spatially extensive events, these values can be interpreted as an upper bound of flood hazards.

Second, we find limited differences in flood hazards across age and education subgroups. Older adults tend to show slightly lower proportion of population living in flood-prone areas, while children (aged < 10 y) show marginally higher shares relative to the total population. The observed patterns are broadly consistent with prior vulnerability literature identifying children in Mexico as particularly susceptible to flood impacts due to limited mobility and dependence on caregivers [33]. For education, most countries (except Mexico and Central America) show slightly higher flood hazards among adults with limited educational attainment compared with total population (Table 1), which may reflect their relatively higher concentration in flood-prone urban areas and is consistent with Kephart et al. [18]. However, these modest differences may also be influenced by aggregation and weighting across countries. In addition, the use of coarse education categories may limit the ability to detect finer gradients in exposure.

Third, we detect significant spatial unevenness in the distribution of flood hazards across most cities, with few residents shouldering substantial exposure to flood hazards. While this concentration may facilitate more targeted mitigation efforts, it also signals that flood risk falls disproportionately on a small and potentially marginalized segment of the population. Of concern are the handful of cities (Balneario Camboriu and Itajai in Brazil) with a *Gini_flood_* lower than 0.75, suggesting that flood hazard is more evenly distributed across residents; these 2 cities also have a relatively high disproportionality in population exposure relative to the share of land in flood hazard areas.

Fourth, we find small but coherent associations between heterogeneity measures and social and environmental variables. Higher *EDI* (greater disproportionality) occurs in denser, steeper, higher elevation, and more topographically rugged inland cities. Such patterns are consistent with our hypotheses and plausibly reflect settlement pressures toward valley bottoms and terrace edges in constrained terrain [34,35]. Higher *Gini_flood_* (greater unevenness) is associated with higher elevation, greater greenspace, and inland location, partially aligning with expectations. The elevation and coastal location patterns in these associations help reconcile the results. Higher inland cities show a slightly greater tendency to place people in flood-prone areas yet spread that exposure across more units. Our results complement the previous study at the neighborhood level by Kephart et al. [18], who report higher odds of historical flooding in neighborhoods characterized by lower educational attainment, coastal location, lower density, greater distance from the city center, greater greenspace, and steeper slopes. While both studies point toward uneven flood burdens, they capture different stages of the hazard-to-impact pathway. Our study measures residential location within modeled riverine flood-prone areas as a proxy for flood susceptibility, whereas Kephart et al. [18] use the Global Flood Database of satellite-observed historical flood events, capturing where damaging floods have actually occurred. Several findings tell complementary stories across scales. Kephart et al. find that greener neighborhoods flood more, likely because vegetation and flood hazard co-occur in floodplains and wetlands where water concentrates. At the city level, we find that greener cities exhibit greater unevenness (higher *Gini_flood_*) but not a higher share of flood-prone population, suggesting that flood risk in these cities is concentrated in fewer areas rather than spread broadly. Similarly, Kephart et al. find flooding in steeper neighborhoods, while we find greater disproportionality in steeper cities, both pointing to terrain as a driver of uneven risk but operating through different mechanisms at different scales. On coastal location, Kephart et al. find more flooding in coastal neighborhoods, whereas we find that coastal cities have higher aggregate exposure, but inland cities display greater disproportionality and unevenness. In other words, realized floods may hit coastal areas more frequently, but the spatial inequality of susceptibility is more pronounced inland. On density, Kephart et al. find flooding in lower-density neighborhoods, while our *EDI* is higher in denser cities. Within cities, low-density peripheries may sit on flooded areas, while across cities, denser settings concentrate more residents near constrained flood-prone areas. In terms of population subgroups, while a larger proportion of adults without primary education reside in flood-prone areas in most countries, our disproportionality measure (*EDI*) indicates that this pattern does not exceed what would be expected given the underlying distribution of flood-prone areas within cities. Yet, Kephart et al. find that such communities still experience a higher burden of realized flood impacts, potentially due to pluvial flooding, proximity to poorly mapped drainage channels, infrastructure deficits, and housing vulnerability. Both studies thus point toward uneven flood burdens, but at different stages of the hazard-to-impact pathway: Our results speak to where riverine flood susceptibility is located, while Kephart et al. highlight where damaging floods have actually occurred and accumulated.

Fifth, *EDI* and *Gini_flood_* reveal distinct patterns that differ from what has been estimated for other regions. In this study, city-level *EDI* is on average slightly negative. This pattern is evident in most countries, but Chile stands out as the only country with a positive median *EDI*, suggesting a tendency toward disproportionate settlement in flood-prone areas. Compared with a previous study in the contiguous United States, which reported an *EDI* of −3.76 [21], Latin American cities exhibit substantially higher relative flood hazard exposure. Subgroup *EDI* largely mirrors those of the total population, with only minor deviations. While most countries exhibit small negative median *EDI*, the spread of values varies considerably, with Colombia showing the largest variation across cities. Mexico contrasts with this general pattern by exhibiting more frequent positive *EDI*, indicating that in some cities, populations are more concentrated in flood-prone areas than the share of land in those areas. This may reflect differences in urban form, settlement patterns, and the spatial relationship between development and river systems. In contrast, *Gini_flood_* values are consistently high across most cities, indicating strong spatial unevenness in the distribution of populations exposed to flood-prone areas. This suggests that even where overall exposure is not disproportionately high (as indicated by *EDI*), exposed populations tend to be concentrated in a limited number of spatial units. Such patterns are consistent with the clustering of urban development along river corridors, which concentrates flood hazard exposure [36]. Compared to *EDI, Gini*_*flood*_ shows less variation across subgroups at the regional scale.

Finally, jointly considering *EDI* and *Gini_flood_* values can provide insights with different implications related to flood risks. Cities with high values for both (Quadrant 1 in Fig. 7) have likelihood of residing in flood-prone areas with clustering of populations in certain flood-prone areas. Risk reduction and protection are recommended to be prioritized for identified hot spots [37]. Cities with high *EDI* and lower *Gini_flood_* (Quadrant 4) have widespread exposure with relatively even spread. City-wide measures should be emphasized, such as land use controls, resilient infrastructure, and housing strategies that reduce exposure at scale [38]. Cities with lower *EDI* and higher *Gini_flood_* (Quadrant II) have lower likelihood of residing in flood-prone areas but high spatial unevenness. Place-based upgrades, such as micro-drainage improvement, and selective buyouts should be targeted [39]. Cities with lower *EDI* and lower *Gini_flood_* (Quadrant III) have a relatively favorable configuration as compared to others. This combined categorization of 2 heterogeneity indices offers a relative assessment for policymakers to address environmental justice issues in flood risk management.

To evaluate the sensitivity of the 2 indices to flood hazard return periods, we calculated *EDI* and *Gini_flood_* across 7 return periods (Figs. S10 and S11 and Tables S4 and S5). Overall, both indices show limited variation across return periods, indicating that patterns of disproportionality and unevenness are largely stable across different flood magnitudes in Latin America. We observe a slight increase in *EDI* from rarer to more frequent floods, suggesting that populations are relatively more concentrated in areas exposed to frequent flooding, while fewer people reside in zones associated with extreme, low-probability events. Similarly, *Gini_flood_* shows a modest increase for more frequent floods, indicating greater spatial unevenness under these conditions. These differences are small, and further work is needed to assess their robustness and underlying drivers.

There are some limitations in this study. First, integrating datasets with different spatial and temporal resolutions can cause information loss and mask fine-scale disparities. Although we apportion population using building footprints, temporal and positional mismatches with census vintages may bias estimates, especially for age-specific measures, which are derived from disaggregated data and may exhibit limited spatial variability at the sub-city scale. Buildings are not only devoted to residential uses, so some population is likely mis-apportioned to non-residential buildings. Similarly, census years differ across countries, which reduces comparability and adds uncertainty for subgroup analyses. Moreover, our socioeconomic status measures are limited to broad population-level indicators, and important dimensions such as race and ethnicity, which are known to be critical for understanding environmental justice in flood risk, could not be incorporated due to lack of consistent data across cities and countries. The statistical modeling used may be improved as more suitable specifications, such as controlling the extent of spatial clustering, are identified. In addition, our analysis focuses on place of residence, but individuals may experience flood exposure in other key contexts of daily life, particularly at their place of work, which is not captured in this study. Second, the JRC flood hazard layer primarily represents riverine flooding and does not explicitly capture pluvial or coastal hazards. Riverine flooding is generally recognized as the predominant source of flood exposure globally, while in our study area only 27% of cities (97 out of 363) are coastal and potentially affected by coastal flooding. Pluvial flooding, on the other hand, remains difficult to quantify consistently due to the lack of regionally standardized monitoring or modeling methods. In addition, the JRC hazard maps do not account for flood control infrastructure in Latin American cities, which may lead to overestimation of population hazards in locations with effective flood-management infrastructure. Third, we assess hazard extent without depth, so the flood-prone area designation indicates riverine susceptibility but cannot be interpreted as how intense the inundation would be. Accordingly, the results support screening and preparedness rather than damage estimation and should be complemented with hazard severity and protection data where available. Fourth, our analysis is intentionally designed as a pattern-oriented descriptive assessment of flood hazard disparities, which is a necessary foundation for future predictive work in this under-studied region. It does not explicitly model the mechanisms generating these patterns, nor does it incorporate probabilistic representations of flood hazard. As such, it does not fully capture nonlinear dynamics, higher-order interactions, or uncertainty propagation underlying observed patterns.

Future work can extend our framework by integrating environmental, infrastructural, and sociodemographic drivers within a systems perspective to disentangle their relative contributions and interactions, which likely shape exposure outcomes through complex dynamics [40]. Building on evidence that environmental patterns are structured by physiographic gradients and network configurations [41,42] future analyses could further quantify these processes and their interactions. In addition, applying global sensitivity and uncertainty analysis would enable systematic identification of dominant drivers and higher-order interactions [43], while causal inference approaches such as convergent cross mapping could help identify directional relationships between flood exposure and sociodemographic patterns [44]. Examining trade-offs among competing urban processes, such as accessibility and flood risk, may also provide additional insight into the emergence of observed disparities, for example, through Pareto-based frameworks [45].

## Conclusion

This study applies indices of disproportionality and unevenness in flood hazard across 363 Latin American cities. We estimate that 6.9% of Latin American urban population live in areas prone to floods with an annual probability of 0.2%. We find substantial spatial heterogeneity: Most countries show slightly negative median *EDI* values, indicating no systematic overconcentration of populations in flood-prone areas, while *Gini_flood_* values are consistently high, reflecting strong spatial clustering of exposed populations. Inland, higher-elevation cities tend to exhibit greater disproportionality and unevenness. Differences across age and education subgroups are limited, and sensitivity analyses suggest that these patterns are stable across return periods. These findings suggest that urban flood hazards are spatially concentrated, supporting targeted, place-based interventions (e.g., zoning and infrastructure) and the integration of finer-scale sociodemographic information for environmental justice considerations. Overall, the proposed framework provides a consistent way to assess relative flood hazards and identify cities where spatial concentration of risk may warrant targeted intervention. Future work should validate these indices, incorporate additional dimensions of heterogeneity, and test robustness across alternative datasets.

## Supplementary Material

Figures, tables, references

## Figures and Tables

**Table 1:** Population and subgroup count by country and the number and percentage of population and subgroups living in flood-prone areas by country in 363 Latin American cities

		Population (in millions) ^a^					
	No. of grid cells (*N*)	Total	<10 y	10–64 y	>64 y	>25 y w/o primary education	>25 y w/ primary education
Overall	1,201,563	297.9	47.4	226.7	23.7	31.1	163.2
By country grouping							
Argentina	276,623	27.8	4.5	20.5	2.9	1.8	14.5
Brazil	371,139	108.9	15.3	85.9	7.7	20.1	45.2
Central America ^b^	10,311	7.0	1.3	5.2	0.4	0.7	2.9
Chile	103,274	12.3	1.6	9.3	1.4	0.7	7.2
Colombia	49,907	26.8	3.5	20.9	2.5	2.1	14.7
Mexico ^c^	346,139	97.4	18.4	71.7	7.4	4.2	69.8
Peru	44,170	17.7	2.8	13.4	1.5	1.5	8.9
		Population (in millions) and percentage of population in flood-prone areas ^d^					
	No. of grid cells (*N*)	Total	<10 y	10–64 y	>64 y	>25 y w/o primary education	>25 y w/ primary education
Overall	1,201,563	20.6 (6.9%)	3.5 (7.3%)	15.5 (6.8%)	1.6 (6.7%)	2.0 (6.3%)	11.1 (6.8%)
By country grouping							
Argentina	276,623	5.5 (19.9%)	0.9 (21.2%)	4.1 (19.9%)	0.5 (17.7%)	0.4 (22.3%)	2.7 (18.9%)
Brazil	371,139	5.7 (5.2%)	0.8 (5.5%)	4.4 (5.2%)	0.4 (5.0%)	1.0 (5.2%)	2.3 (5.0%)
Central America ^b^	10,311	0.1 (1.1%)	0.0 (1.1%)	0.1 (1.1%)	0.0 (1.1%)	0.0 (0.6%)	0.0 (1.3%)
Chile	103,274	0.3 (2.8%)	0.0 (2.9%)	0.3 (2.8%)	0.0 (2.7%)	0.0 (3.2%)	0.2 (2.7%)
Colombia	49,907	1.6 (5.9%)	0.2 (6.7%)	1.2 (6.0%)	0.1 (4.0%)	0.1 (6.0%)	0.8 (5.4%)
Mexico ^c^	346,139	6.7 (6.9%)	1.3 (6.9%)	4.9 (6.9%)	0.5 (7.0%)	0.3 (7.2%)	4.8 (6.9%)
Peru	44,170	0.7 (3.9%)	0.1 (4.6%)	0.5 (3.8%)	0.0 (3.3%)	0.1 (4.6%)	0.3 (3.4%)

a Population is in millions and is apportioned to grid cells from census units based on building footprints. Country statistics are aggregated from grid cells overlapping with cities in each country. See Eq. (1) to (4) for details.

b Central America includes cities in the countries of Guatemala (*n* = 3), Costa Rica (*n* = 1), and Panama (*n* = 3).

c Mexico uses years >15 instead of 25 in education attainment due to data availability.

d The population percentage in flood-prone areas is calculated by dividing the subgroup count in flood-prone areas by the total for that subgroup in each country.

## Data Availability

Population and census data for Brazil, Chile, and Mexico were downloaded from publicly available repositories from statistical agencies in each country. Population and census data for Argentina, Costa Rica, El Salvador, Guatemala, Panama, and Peru were obtained directly from statistical agencies in each country. A link to these agency websites can be accessed via https://drexel.edu/lac/data-evidence/data-acknowledgements/. Urban boundaries and features obtained from the SALURBAL-Climate project are freely available at https://data.lacurbanhealth.org/. Please contact the corresponding author about access to any urban features that are not yet published on the SALURBAL-Climate portal. JRC flood hazard maps, which provide global river flood inundation depth at ~90-m resolution for 7 return periods (10 to 500 y), are available at https://jeodpp.jrc.ec.europa.eu/ftp/jrc-opendata/CEMS-GLOFAS/flood_hazard/. The code used to process and harmonize these data is available at https://github.com/jinwenxu/flood-heterogeneity-in-latin-america.
