# Supplementary material for: Heterogeneity in Flood Hazard Exposure in 363 Latin American Cities
Source: Ecosyst Health Sustain. Author manuscript; Available in PMC 2026 Aug 16. (PMC7619367; doi:10.34133/ehs.0567)
Supplement: Supplementary file 1 [file EMS217150-supplement-Figures__tables__references.docx]

SUPPLEMENTARY MATERIALS

**Supplementary Appendix I**. Detailed descriptions of urban environmental measures and their interpretation

**Population**: Number of residents from the most recent national census (see Supplementary Table S1 for census year by country).

**Population growth**: The 5-year growth as percent of population change from 2015 to 2020

**Population density**: Residents (in thousands) per square kilometer of neighborhood/city area, based on national census data.

**Intersection density**: Number of intersections per square kilometer of built-up area, derived from OpenStreetMap street networks. Intersections are defined as nodes with >2 connecting streets (cul-de-sacs and bends excluded). Neighborhood and city indices correspond to 2020 and 2017, respectively.

**Gross Domestic Product per capita**: City-level GDP in 2011 international US dollars, derived from Gennaioli et al. (2013) [46] and spatialized by Kummu et al. (2018) [47]. The gridded dataset covers 1990–2015 and combines government, survey, and industry data. GDP values were matched to SALURBAL cities using administrative boundaries or population-weighted averages for cities spanning multiple units. Estimates from 2015 (the latest available year) were used.

**Average elevation**: Mean land elevation (meters above sea level), derived from the Shuttle Radar Topography Mission (SRTM) 30 m global elevation dataset [48]. Permanent water bodies were excluded using the JRC Yearly Water Classification History dataset [49].

**Slope**: Mean land slope (degrees), calculated from the SRTM elevation model using local elevation gradients. Higher values indicate steeper terrain. Water bodies were excluded as above.

**Slope standard deviation**: Variation in slope values across land area (degrees), representing terrain ruggedness. Larger values indicate more heterogeneous or uneven topography. See Keskinen et al. (2018) [50].

**Greenness**: Area-level median greenness measured by the normalized difference vegetation index (NDVI). NDVI was derived from the MODIS vegetation product MOD13Q1.006 (250 m resolution). For each grid cell, the maximum NDVI in 2019 was used to capture peak greenness, and neighborhood medians were then computed.

**Coastal location**: Cities with boundaries within 1 km of the nearest ocean coastline, based on the Global Self-consistent, Hierarchical, High-resolution Geography Database (GSHHG, high-resolution version). All others are classified as inland.

**Fig. S1.** City-level scatterplot of the proportion of population in flood hazard zones against the share of city land classified as flood-prone (excluding permanent water). Axes are square-root scaled to improve visibility in the lower range. The diagonal line indicates equal proportions (*EDI* = 0), with points above the line representing cities where population exposure exceeds land share (*EDI* > 0).

**Table S1.** Administrative boundaries and Census year, and number of 1km grid cells in each country

| **Country Name** | **Administrative Unit** | **Census Year** | **Number of Cities** | **Number of 1km grid cells** |
| --- | --- | --- | --- | --- |
| Argentina | Fraccion Censal | 2010 | 33 | 276,623 |
| Brazil | Áreas de Ponderação | 2010 | 152 | 371,139 |
| Chile | Zona Censal | 2017 | 21 | 103,274 |
| Colombia | Sector Urbano, Clase = 1 | 2018 | 35 | 49,907 |
| Mexico | Basic Geostatistical Area (AGEB) | 2020 | 92 | 346,139 |
| Peru | Zona Censal | 2017 | 23 | 44,170 |
| Central America^*^ |  |  |  | 10,311 |
| Costa Rica | Distrito | 2011 | 1 | 2,796 |
| Guatemala | Sector Censal | 2002 | 3 | 2,445 |
| Panama | Barrio | 2010 | 3 | 5,070 |

^*^ Due to the limited number of cities in each country, we aggregated them under the regional label **Central America**, which includes Guatemala (n = 3), Costa Rica (n = 1), and Panama (n = 3).

**Fig. S2.** Pairwise mean differences in *EDI* across country/region pairs, with 95% bootstrap confidence intervals (5,000 resamples). Red markers indicate pairs whose interval excludes zero; grey markers indicate non-significant pairs.

**Fig. S3.** The distribution of *EDI* in (a) total population (Total), (b) children aged less than 10 years (< 10 yrs old), (c) young and mid-age people (10-64 yrs old), (d)adults aged 64+ years (>64 yrs old), (e) adults aged 25+ who completed primary education or more (PriEdu), and (f) adults aged 25+ who did not complete primary education (NoPriEdu)

**Table S2**. The number of cities that have a positive or negative *EDI* in total population and subgroups and are significantly different from *EDI* of spatial random population distribution (percentages to the total number of cities analyzed are included in the parenthesis).

|  | **N** | **EDI_total_** | | | **EDI_< 10 yrs old_** | | | **EDI_10-64 yrs old_** | | | **EDI_> 64 yrs old_** | | | | **EDI_adults w/  primary education_** | | | | **EDI_adults w/o  primary education_** | | |
| --- | --- | --- | --- | --- | --- | --- | --- | --- | --- | --- | --- | --- | --- | --- | --- | --- | --- | --- | --- | --- | --- |
|  |  | **Sig. & Pos.** | **Sig. & Neg.** | **Non-sig.** | **Sig. & Pos.** | **Sig. & Neg.** | **Non-sig.** | **Sig. & Pos.** | **Sig. & Neg.** | **Non-sig.** | **Sig. & Pos.** | **Sig. & Neg.** | **Non-sig.** | **Sig. & Pos.** | | **Sig. & Neg.** | **Non-sig.** | **Sig. & Pos.** | | **Sig. & Neg.** | **Non-sig.** |
| Overall | 363 | 70  (19%) | 88  (24%) | 205  (56%) | 65  (18%) | 79  (22%) | 219  (60%) | 65  (18%) | 91  (25%) | 207  (57%) | 66  (18%) | 83  (23%) | 214  (59%) | 64  (18%) | | 90  (25%) | 209  (58%) | 64  (18%) | | 91  (25%) | 208  (57%) |
| Argentina | 33 | 10  (30%) | 5  (15%) | 18  (55%) | 8  (24%) | 3  (9%) | 22  (67%) | 9  (27%) | 4  (12%) | 20  (61%) | 10  (30%) | 4  (12%) | 19  (58%) | 8  (24%) | | 4  (12%) | 21  (64%) | 9  (27%) | | 4  (12%) | 20  (61%) |
| Brazil | 152 | 34  (22%) | 40  (26%) | 78  (51%) | 34  (22%) | 38  (25%) | 80  (53%) | 34  (22%) | 42  (28%) | 76  (50%) | 34  (22%) | 41  (27%) | 77  (51%) | 35  (23%) | | 40  (26%) | 77  (51%) | 34  (22%) | | 42  (28%) | 76  (50%) |
| Central America | 7 | 0  (0%) | 3  (43%) | 4  (57%) | 0  (0%) | 2  (29%) | 5  (71%) | 0  (0%) | 3  (43%) | 4  (57%) | 0  (0%) | 2  (29%) | 5  (71%) | 0  (0%) | | 3  (43%) | 4  (57%) | 0  (0%) | | 3  (43%) | 4  (57%) |
| Chile | 21 | 7  (33%) | 3  (14%) | 11  (52%) | 4  (19%) | 3  (14%) | 14  (67%) | 6  (29%) | 3  (14%) | 12  (57%) | 5  (24%) | 3  (14%) | 13  (62%) | 6  (29%) | | 3  (14%) | 12  (57%) | 6  (29%) | | 3  (14%) | 12  (57%) |
| Colombia | 35 | 3  (9%) | 5  (14%) | 27  (77%) | 1  (3%) | 5  (14%) | 29  (83%) | 3  (9%) | 5  (14%) | 27  (77%) | 2  (6%) | 5  (14%) | 28  (80%) | 1  (3%) | | 5  (14%) | 29  (83%) | 2  (6%) | | 5  (14%) | 28  (80%) |
| Mexico | 92 | 11  (12%) | 26  (28%) | 55  (60%) | 15  (16%) | 24  (26%) | 53  (58%) | 10  (11%) | 28  (30%) | 54  (59%) | 11  (12%) | 23  (25%) | 58  (63%) | 11  (12%) | | 29  (32%) | 52  (57%) | 10  (11%) | | 28  (30%) | 54  (59%) |
| Peru | 23 | 5  (22%) | 6  (26%) | 12  (52%) | 3  (13%) | 4  (17%) | 16  (70%) | 3  (13%) | 6  (26%) | 14  (61%) | 4  (17%) | 5  (22%) | 14  (61%) | 3  (13%) | | 6  (26%) | 14  (61%) | 3  (13%) | | 6  (26%) | 14  (61%) |

**Table S3.** Grid cells and population exposed to flood-prone areas, by grid-cell education attainment and age groups

|  | **# grid cells (N)** | **Total population**  **(millions)** | **Flood-prone population (area-weighted, millions)** | **% Flood-prone population (area-weighted)** | **Population in exposed grid cells (binary, millions)** | **% Population in exposed grid cells (binary)** |
| --- | --- | --- | --- | --- | --- | --- |
| **Summary** | 450296 | 297.9 | 20.6 | 6.9 | 66.2 | 22.2 |
| **By grid-cell %older adults (aged 64+)** | | | | | | |
| 1st quintile (lowest) | 90067 | 75.1 | 5.0 | 6.7 | 15.2 | 20.2 |
| 2nd quintile | 90575 | 53.1 | 3.9 | 7.3 | 12.4 | 23.4 |
| 3rd quintile | 89632 | 41.8 | 2.7 | 6.4 | 9.1 | 21.9 |
| 4th quintile | 89963 | 49.5 | 3.5 | 7.1 | 11.2 | 22.6 |
| 5th quintile (highest) | 90059 | 78.5 | 5.5 | 7.0 | 18.3 | 23.3 |
| **By grid-cell %children (aged < 10)** | | | | | | |
| 1st quintile (lowest) | 90095 | 111.2 | 5.6 | 5.0 | 23.7 | 21.3 |
| 2nd quintile | 90169 | 68.1 | 5.0 | 7.4 | 16.3 | 24.0 |
| 3rd quintile | 89914 | 48.9 | 3.6 | 7.4 | 11.4 | 23.3 |
| 4th quintile | 90252 | 39.9 | 4.2 | 10.5 | 9.5 | 23.8 |
| 5th quintile (highest) | 89866 | 29.8 | 2.1 | 7.2 | 5.3 | 17.8 |
| **By grid-cell %adults aged 25+ who completed primary education or more** | | | | | | |
| 1st quintile (lowest) | 90060 | 4.5 | 0.3 | 7.3 | 0.8 | 17.9 |
| 2nd quintile | 90062 | 27.6 | 1.7 | 6.2 | 6.4 | 23.2 |
| 3rd quintile | 90056 | 67.1 | 5.0 | 7.5 | 15.9 | 23.8 |
| 4th quintile | 90750 | 82.2 | 6.0 | 7.3 | 20.0 | 24.4 |
| 5th quintile (highest) | 89368 | 116.7 | 7.6 | 6.5 | 23.1 | 19.8 |

**Fig. S4.** Distribution of city-level flood-prone proportions for significant versus non-significant *EDI*, with overlaid kernel density curves indicating distributional trends.

**Fig. S5.** The distribution of *Gini_flood_* in (a) total population (Total), (b) children aged less than 10 years (< 10 yrs old), (c) young and mid-age people (10-64 yrs old), (d)adults aged 64+ years (>64 yrs old), (e) adults aged 25+ who completed primary education or more (PriEdu), and (f) adults aged 25+ who did not complete primary education (NoPriEdu)

**Fig. S6.** Pairwise mean differences in *Gini_flood_* across country/region pairs for six population subgroups: (A) total population, (B) children under 10 years, (C) ages 10–64, (D) adults over 64, (E) adults who completed primary education, and (F) adults who did not complete primary education. Each panel shows 95% bootstrap confidence intervals (5,000 resamples) for pairwise mean differences. Red markers indicate pairs whose interval excludes zero; grey markers indicate non-significant pairs. Kruskal–Wallis test results and the number of significant pairs are annotated in each panel.

**Fig. S7.** Grouped violin plots showing the relationships between the proportion of flood-prone population in subgroups and a range of socioeconomic and environmental variables across cities. Each cell displays the Spearman correlation coefficient (ρ), ρ with significant correlations (*p*<0.05) highlighted in blue and bold font.

**Fig. S8.** Grouped violin plots showing the relationships between the Exposure Deviational Index (*EDI*) in subgroups and a range of socioeconomic and environmental variables across cities. Each cell displays the Spearman correlation coefficient (ρ), ρ with significant correlations (*p*<0.05) highlighted in blue and bold font.

**Fig. S9.** Grouped violin plots showing the relationships between the *Gini_flood_* Index in subgroups and a range of socioeconomic and environmental variables across cities. Each cell displays the Spearman correlation coefficient (ρ), ρ with significant correlations (*p*<0.05) highlighted in blue and bold font.

**Fig. S10**. Sensitivity analysis of *EDI* under different magnitudes of floods represented by return periods, e.g., 500-year floods are floods with annual probability at 0.2% (rare and large floods), 10-year floods are floods with annual probability at 10% (more common and small floods).

**Table S4**. Distribution of Exposure Deviation Index (*EDI*) across return periods (N = 363 cities)

| **Return period** | **Mean** | **SD** | **Median** | **Min** | **Max** | **% Positive** | **% Negative** |
| --- | --- | --- | --- | --- | --- | --- | --- |
| 10-Year | 0.011 | 0.107 | -0.004 | -0.398 | 0.627 | 35.8 | 63.9 |
| 20-Year | 0.006 | 0.107 | -0.006 | -0.412 | 0.606 | 33.9 | 65.8 |
| 50-Year | 0.001 | 0.107 | -0.007 | -0.425 | 0.586 | 31.4 | 68.3 |
| 75-Year | 0 | 0.107 | -0.008 | -0.43 | 0.576 | 30.6 | 69.1 |
| 100-Year | -0.002 | 0.107 | -0.008 | -0.434 | 0.568 | 30.3 | 69.4 |
| 200-Year | -0.005 | 0.107 | -0.009 | -0.442 | 0.55 | 28.7 | 71.1 |
| 500-Year | -0.008 | 0.107 | -0.009 | -0.452 | 0.531 | 27.3 | 72.5 |

**Fig. S11**. Sensitivity analysis of *Gini_flood_* under different magnitudes of floods represented by return periods, e.g., 500-year floods are floods with annual probability at 0.2% (rare and large floods), 10-year floods are floods with annual probability at 10% (more common and small floods).

**Table S5**. Distribution of *Gini_flood_* across return periods (N = 361 cities)

| **Return period** | **Mean** | **SD** | **Median** | **Min** | **Max** |
| --- | --- | --- | --- | --- | --- |
| 10-Year | 0.974 | 0.033 | 0.985 | 0.739 | 1 |
| 20-Year | 0.973 | 0.036 | 0.984 | 0.72 | 1 |
| 50-Year | 0.971 | 0.038 | 0.984 | 0.705 | 1 |
| 75-Year | 0.971 | 0.039 | 0.983 | 0.687 | 1 |
| 100-Year | 0.97 | 0.04 | 0.983 | 0.676 | 1 |
| 200-Year | 0.969 | 0.041 | 0.982 | 0.659 | 1 |
| 500-Year | 0.968 | 0.043 | 0.981 | 0.637 | 1 |
